# Bis(acetyl­acetonato-κ^2^ *O*,*O*′)­aqua­(diacetyl­methanido-κ*C*)iridium(III)

**DOI:** 10.1107/S160053680903760X

**Published:** 2009-09-30

**Authors:** Qiao-Wen Chang, Ming-Jin Xie, Wei-Ping Liu, Xi-Zhu Chen, Qing-Song Ye

**Affiliations:** a Chemistry Laboratory, Kunming Institute of Precious Metals, Kunming, People’s Republic of China; b Department of Chemistry, Yunnan University, Kunming, People’s Republic of China

## Abstract

In the crystal structure of the title compound, [Ir(C_5_H_7_O_2_)_3_(H_2_O)], the Ir^III^ atom is six-coordinated and situated in a slightly distorted octa­hedral environment. The complex contains both Ir—O and Ir—C bonds and was isolated from a reaction mixture of IrCl_3_(H_2_O)_*x*_, pentane-2,5-dione and NaHCO_3_. O—H⋯O hydrogen bonding between the water molecules and the carbonyl O atoms of adjacent molecules leads to a layered motif extending parallel to (010).

## Related literature

For background to the title compound, see: Bennett & Mitchell (1976 ▶); Bhalla *et al.* (2005 ▶); Gibson (1969 ▶); Matsumoto *et al.* (2000 ▶); Periana *et al.* (2002 ▶); Wong-Foy *et al.* (2003 ▶). For a related structure, see: Isakova *et al.* (1999 ▶); For background on hydrogen bonding, see: Desiraju (1996 ▶). 
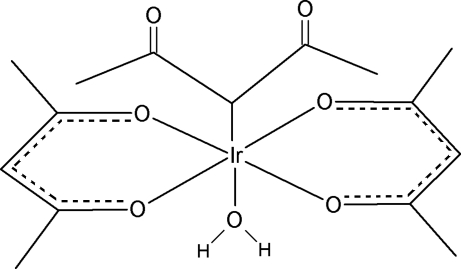


## Experimental

### 

#### Crystal data

[Ir(C_5_H_7_O_2_)_3_(H_2_O)]
*M* *_r_* = 507.54
Triclinic, 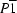

*a* = 7.7853 (13) Å
*b* = 7.9461 (13) Å
*c* = 16.362 (3) Å
α = 77.295 (2)°
β = 77.927 (2)°
γ = 60.918 (1)°
*V* = 856.7 (3) Å^3^
*Z* = 2
Mo *K*α radiation
μ = 7.82 mm^−1^
*T* = 293 K
0.15 × 0.13 × 0.09 mm

#### Data collection

Bruker APEXII CCD area-detector diffractometer
Absorption correction: multi-scan (*SADABS*; Sheldrick, 1996 ▶) *T*_min_ = 0.387, *T*_max_ = 0.539
7435 measured reflections
3900 independent reflections
2885 reflections with *I* > 2σ(*I*)
*R*_int_ = 0.061

#### Refinement

*R*[*F* ^2^ > 2σ(*F* ^2^)] = 0.058
*wR*(*F* ^2^) = 0.127
*S* = 1.03
3900 reflections
215 parameters
H-atom parameters constrained
Δρ_max_ = 2.37 e Å^−3^
Δρ_min_ = −1.88 e Å^−3^

### 

Data collection: *APEX2* (Bruker, 2004 ▶); cell refinement: *SAINT* (Bruker, 2004 ▶); data reduction: *SAINT*; program(s) used to solve structure: *SHELXS97* (Sheldrick, 2008 ▶); program(s) used to refine structure: *SHELXL97* (Sheldrick, 2008 ▶); molecular graphics: *SHELXTL* (Sheldrick, 2008 ▶); software used to prepare material for publication: *SHELXTL*.

## Supplementary Material

Crystal structure: contains datablocks global, I. DOI: 10.1107/S160053680903760X/er2069sup1.cif

Structure factors: contains datablocks I. DOI: 10.1107/S160053680903760X/er2069Isup2.hkl

Additional supplementary materials:  crystallographic information; 3D view; checkCIF report

## Figures and Tables

**Table 1:** Selected bond lengths (Å)

Ir1—O1	2.015 (8)
Ir1—O4	2.017 (7)
Ir1—O3	2.026 (7)
Ir1—O2	2.029 (7)
Ir1—C11	2.131 (11)
Ir1—O5	2.155 (7)

**Table 2:** Hydrogen-bond geometry (Å, °)

*D*—H⋯*A*	*D*—H	H⋯*A*	*D*⋯*A*	*D*—H⋯*A*
O5—H5*D*⋯O7^i^	0.82	2.24	2.706 (11)	116
O5—H5*E*⋯O6^ii^	0.89	2.11	2.715 (11)	125

Symmetry codes: (i) 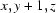; (ii) 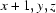.

**Fig. 1.**
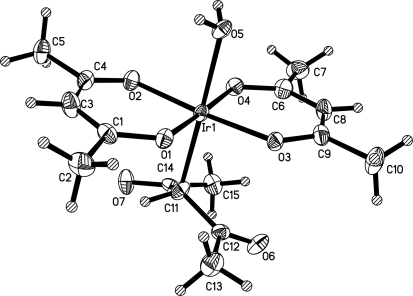
Molecular view of the complex, with the atomic labeling scheme. Displacement ellipsoids are shown at the 30% probability level.

**Fig. 2.**
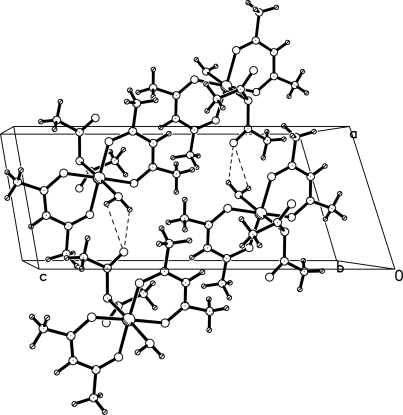
Packing view, showing the C—H···O hydrogen-bond network between the complexes. Only the H atoms involved in H bonding are drawn. Hydrogen bonds are shown as dashed lines.

## supplementary crystallographic information

### Comment

[Ir(acac-O, O)_2_(acac-C^3^)(H_2_O)], as a novel, themally stable, homogeneous bis-acac-O, O—Ir(III) complex catalyst for the anti-Markovnikov, hydroarylation of olefins, has attracted considerable attention (Periana *et al.*, 2002, Wong-Foy *et al.* 2003, Bhalla *et al.*, 2005). Bennett *et al.* obtained [Ir(µ-acac-O,*O*,*C*^3^)(acac-O, O)(acac-C^3^)] and an unknown yellow solid as byproducts in the course of an attempt to improve the synthesis of Ir(acac)_3_ (Bennett & Mitchell, 1976). However, they were unable to purify the yellow solid or characterize its structure. Recently Periana *et al.* successfully isolated this yellow product in pure form and proved it to be [Ir(acac-O, O)_2_(acac-C^3^)(H_2_O)] by spectroscopic data(Periana *et al.*, 2002). Despite the thermal and oxidative stability of octahedral iridium (III)-carbon σ -bonded complexes, there are little well characterized γ-C-bonded β-diketone complexes of iridium (III) except [Ir(µ-acac-O,*O*,*C*^3^)(acac-O, O)(acac-C^3^)] (Gibson, 1969, Matsumoto *et al.*, 2000).

Recently the single-crystal of the compound was obtained in our laboratory and we report its crystallographic structure. In the complex, the central Ir atom is coordinated by a octahedron of four oxygen atoms of two acetylacetone ligands, one carbon atom of one acetylacetone ligand and one oxygen atom of water molcule. The average Ir—O bond length of two acetylacetone ligands is 2.02 Å, which agree with the literature data of Ir(acac)_3_ (Isakova *et al.*, 1999), however, Ir—O bond length of coordinated water is 2.15 Å, which is longer than the former, whereas Ir—C bond length is found to be 2.13 Å. Intermolecular C—H···O hydrogen bond (Desiraju, 1996) and C—H···C hydrogen bond are present in the structure; while the C···O distances are within the range of 3.039 (9)–3.486 (10) Å, C—H···O are found to be within the range of 110–168°. The compound forms a hydrogen-bonded chain in which the O5 water uses two H atom to serve as a donor to the O atoms of adjacent molecules, these weaker interactions giving rise to a three-dimensional framework structure.

### Experimental

The complex was synthesized according literature (see Periana *et al.*, 2002). In a round-bottom flask equipped with a reflux condenser vented to an oil bubble, 5 g of IrCl_3_ (H_2_O)*_x_* (54.34% of Ir, 14.13 mmol), 50 ml of 2, 5-pentadione (48.75 mmol) and 5 g of NaHCO_3_(59.5 mmol) were added. The mixture was heated to gentle reflux with stirring for 48 h. During this time, a yellow solid precipitated. The reaction mixture was cooled to room temperature and the solid was collected as crude product. The yellow solid was dissolved in 200 ml H2O at room temperature under vigorous stirring and was filtered. Solution was concentrated under vacuo to give 3.15 g (43.6%) complex as yellow microcrystalline. The compound was crystallized from water to obtain crystals suitable for X-ray structure analysis. 1HNMR (CD3OD,p.p.m.):5.54(s, 2H, CH), 5.49(s, 1H, CH), 1.96(s, 12H, CH3), 1.83(s, 6H, CH3). 13CNMR (dmso,p.p.m.): 208.4(s, C-acac, C=O), 186.1(s, O-acac, C=O), 102.6(s, O-acac, CH), 48.6(s, C-acac, CH), 31.6(s, C-acac, CH3, 26.6(s, O-acac, CH3). Anal. Calcd: Ir, 37.95; C, 35.54; H, 4.93. Found: Ir, 37.90; C, 35.48; H, 4.87.

### Refinement

All H atoms were initially located in a difference Fourier map but were positioned with idealized geometry and refined isotropic with *U*_iso_(H)=1.2*U*_eq_(C) (1.5 for methyl H atoms) using a riding model with C—H = 0.93 and 0.97 Å).

### Crystal data

**Table:**

[Ir(C_5_H_7_O_2_)_3_(H_2_O)]	*Z* = 2
*M**_r_* = 507.54	*F*(000) = 492
Triclinic, *P*1	*D*_x_ = 1.968 Mg m^−^^3^
*a* = 7.7853 (13) Å	Mo *K*α radiation, λ = 0.71073 Å
*b* = 7.9461 (13) Å	Cell parameters from 1164 reflections
*c* = 16.362 (3) Å	θ = 1.3–28.4°
α = 77.295 (2)°	µ = 7.82 mm^−^^1^
β = 77.927 (2)°	*T* = 293 K
γ = 60.918 (1)°	Block, orange
*V* = 856.7 (3) Å^3^	0.15 × 0.13 × 0.09 mm

### Data collection

**Table:**

Bruker APEXII CCD area-detector diffractometer	3900 independent reflections
Radiation source: fine-focus sealed tube	2885 reflections with *I* > 2σ(*I*)
graphite	*R*_int_ = 0.061
φ and ω scans	θ_max_ = 28.4°, θ_min_ = 1.3°
Absorption correction: multi-scan (*SADABS*; Sheldrick, 1996)	*h* = −10→10
*T*_min_ = 0.387, *T*_max_ = 0.539	*k* = −10→10
7435 measured reflections	*l* = −21→21

### Refinement

**Table:**

Refinement on *F*^2^	Secondary atom site location: difference Fourier map
Least-squares matrix: full	Hydrogen site location: inferred from neighbouring sites
*R*[*F*^2^ > 2σ(*F*^2^)] = 0.058	H-atom parameters constrained
*wR*(*F*^2^) = 0.127	*w* = 1/[σ^2^(*F*_o_^2^) + (0.0469*P*)^2^] where *P* = (*F*_o_^2^ + 2*F*_c_^2^)/3
*S* = 1.02	(Δ/σ)_max_ = 0.001
3900 reflections	Δρ_max_ = 2.37 e Å^−^^3^
215 parameters	Δρ_min_ = −1.87 e Å^−^^3^
0 restraints	Extinction correction: *SHELXL97* (Sheldrick, 2008), Fc^*^=kFc[1+0.001xFc^2^λ^3^/sin(2θ)]^-1/4^
Primary atom site location: structure-invariant direct methods	Extinction coefficient: 0.0013 (5)

### Special details

**Table:**

Geometry. All e.s.d.'s (except the e.s.d. in the dihedral angle between two l.s. planes)are estimated using the full covariance matrix. The cell e.s.d.'s are takeninto account individually in the estimation of e.s.d.'s in distances, anglesand torsion angles; correlations between e.s.d.'s in cell parameters are onlyused when they are defined by crystal symmetry. An approximate (isotropic)treatment of cell e.s.d.'s is used for estimating e.s.d.'s involving l.s. planes.
Refinement. Refinement of *F*^2^ against ALL reflections. The weighted *R*-factor *wR* and goodness of fit *S* are based on *F*^2^, conventional *R*-factors *R* are based on *F*, with *F* set to zero for negative *F*^2^. The threshold expression of *F*^2^ > σ(*F*^2^) is used only for calculating *R*-factors(gt) *etc*. and is not relevant to the choice of reflections for refinement. *R*-factors based on *F*^2^ are statistically about twice as large as those based on *F*, and *R*- factors based on ALL data will be even larger.

### Fractional atomic coordinates and isotropic or equivalent isotropic displacement parameters (Å^2^)

**Table:**

	*x*	*y*	*z*	*U*_iso_*/*U*_eq_	
Ir1	0.36783 (6)	0.62096 (6)	0.25137 (3)	0.02612 (17)	
O1	0.3350 (11)	0.7800 (11)	0.1361 (5)	0.0327 (18)	
O2	0.6421 (11)	0.4122 (11)	0.2154 (5)	0.0355 (19)	
O3	0.1042 (10)	0.8433 (10)	0.2871 (5)	0.0315 (17)	
O4	0.4064 (11)	0.4697 (11)	0.3682 (5)	0.0334 (18)	
O5	0.5066 (10)	0.7689 (10)	0.2858 (5)	0.0337 (18)	
H5D	0.5577	0.8115	0.2430	0.050*	
H5E	0.5997	0.6857	0.3187	0.050*	
O6	−0.0934 (12)	0.5794 (12)	0.2823 (6)	0.046 (2)	
O7	0.4419 (13)	0.1349 (12)	0.2185 (5)	0.048 (2)	
C1	0.4627 (18)	0.7249 (17)	0.0700 (7)	0.035 (3)	
C2	0.402 (2)	0.8478 (19)	−0.0103 (7)	0.047 (3)	
H2A	0.3312	0.8038	−0.0347	0.070*	
H2B	0.5167	0.8398	−0.0483	0.070*	
H2C	0.3172	0.9801	−0.0006	0.070*	
C3	0.6452 (19)	0.5594 (18)	0.0726 (8)	0.046 (3)	
H3	0.7256	0.5384	0.0212	0.055*	
C4	0.7252 (17)	0.4194 (15)	0.1418 (8)	0.034 (3)	
C5	0.9347 (19)	0.256 (2)	0.1293 (9)	0.055 (4)	
H5A	0.9385	0.1353	0.1578	0.083*	
H5B	1.0224	0.2819	0.1519	0.083*	
H5C	0.9756	0.2480	0.0701	0.083*	
C6	0.2844 (19)	0.5342 (18)	0.4321 (7)	0.040 (3)	
C7	0.334 (2)	0.402 (2)	0.5146 (8)	0.055 (4)	
H7A	0.2550	0.3352	0.5289	0.083*	
H7B	0.3074	0.4781	0.5582	0.083*	
H7C	0.4718	0.3091	0.5092	0.083*	
C8	0.1094 (19)	0.7063 (19)	0.4332 (8)	0.044 (3)	
H8	0.0380	0.7318	0.4860	0.052*	
C9	0.0265 (18)	0.8459 (17)	0.3658 (7)	0.036 (3)	
C10	−0.1719 (19)	1.0193 (19)	0.3801 (8)	0.054 (4)	
H10A	−0.1759	1.1296	0.3405	0.080*	
H10B	−0.1923	1.0475	0.4365	0.080*	
H10C	−0.2742	0.9916	0.3725	0.080*	
C11	0.2348 (16)	0.4738 (15)	0.2148 (8)	0.034 (3)	
H11	0.2784	0.4669	0.1544	0.041*	
C12	0.0184 (17)	0.5990 (16)	0.2220 (8)	0.033 (3)	
C13	−0.0656 (19)	0.7536 (19)	0.1483 (8)	0.051 (3)	
H13A	−0.0591	0.6933	0.1022	0.076*	
H13B	0.0101	0.8241	0.1309	0.076*	
H13C	−0.2009	0.8417	0.1649	0.076*	
C14	0.3079 (17)	0.2716 (16)	0.2559 (7)	0.030 (2)	
C15	0.226 (2)	0.2179 (18)	0.3450 (8)	0.046 (3)	
H15A	0.3341	0.1330	0.3776	0.070*	
H15B	0.1511	0.1529	0.3428	0.070*	
H15C	0.1427	0.3337	0.3707	0.070*	

### Atomic displacement parameters (Å^2^)

**Table:**

	*U*^11^	*U*^22^	*U*^33^	*U*^12^	*U*^13^	*U*^23^
Ir1	0.0211 (2)	0.0223 (2)	0.0273 (2)	−0.00479 (17)	−0.00288 (16)	−0.00175 (15)
O1	0.028 (4)	0.031 (4)	0.035 (4)	−0.012 (4)	0.001 (4)	−0.007 (4)
O2	0.030 (4)	0.030 (4)	0.039 (5)	−0.008 (4)	0.000 (4)	−0.006 (4)
O3	0.025 (4)	0.027 (4)	0.032 (4)	−0.002 (3)	−0.007 (3)	−0.007 (3)
O4	0.033 (4)	0.041 (5)	0.024 (4)	−0.017 (4)	−0.004 (3)	0.003 (3)
O5	0.021 (4)	0.032 (4)	0.038 (4)	−0.008 (3)	−0.006 (3)	0.003 (4)
O6	0.026 (5)	0.044 (5)	0.058 (6)	−0.010 (4)	−0.005 (4)	0.000 (4)
O7	0.056 (6)	0.028 (4)	0.048 (5)	−0.011 (4)	0.009 (4)	−0.016 (4)
C1	0.045 (7)	0.039 (7)	0.024 (6)	−0.021 (6)	−0.007 (5)	−0.003 (5)
C2	0.059 (9)	0.051 (8)	0.030 (7)	−0.028 (7)	−0.018 (6)	0.011 (6)
C3	0.052 (8)	0.043 (8)	0.029 (7)	−0.012 (7)	−0.003 (6)	−0.004 (6)
C4	0.030 (6)	0.020 (5)	0.049 (7)	−0.009 (5)	0.004 (6)	−0.015 (5)
C5	0.039 (8)	0.054 (9)	0.060 (9)	−0.010 (7)	0.011 (7)	−0.029 (7)
C6	0.046 (8)	0.048 (8)	0.029 (6)	−0.029 (7)	0.002 (6)	0.002 (6)
C7	0.054 (9)	0.078 (11)	0.039 (7)	−0.036 (8)	−0.011 (7)	0.002 (7)
C8	0.044 (8)	0.055 (8)	0.031 (7)	−0.023 (7)	0.009 (6)	−0.016 (6)
C9	0.039 (7)	0.033 (7)	0.036 (7)	−0.017 (6)	0.003 (6)	−0.012 (5)
C10	0.038 (8)	0.049 (8)	0.043 (8)	0.001 (7)	−0.001 (6)	−0.005 (7)
C11	0.021 (6)	0.022 (6)	0.050 (7)	−0.002 (5)	−0.005 (5)	−0.003 (5)
C12	0.030 (6)	0.026 (6)	0.043 (7)	−0.010 (5)	−0.011 (6)	−0.009 (5)
C13	0.043 (8)	0.047 (8)	0.049 (8)	−0.015 (7)	−0.006 (6)	0.003 (6)
C14	0.038 (7)	0.030 (6)	0.032 (6)	−0.021 (5)	−0.009 (5)	−0.006 (5)
C15	0.058 (9)	0.041 (7)	0.042 (7)	−0.024 (7)	−0.003 (6)	−0.005 (6)

### Geometric parameters (Å, °)

**Table:**

Ir1—O1	2.015 (8)	C5—H5C	0.9600
Ir1—O4	2.017 (7)	C6—C8	1.384 (17)
Ir1—O3	2.026 (7)	C6—C7	1.508 (16)
Ir1—O2	2.029 (7)	C7—H7A	0.9600
Ir1—C11	2.131 (11)	C7—H7B	0.9600
Ir1—O5	2.155 (7)	C7—H7C	0.9600
O1—C1	1.297 (13)	C8—C9	1.388 (17)
O2—C4	1.245 (13)	C8—H8	0.9300
O3—C9	1.302 (13)	C9—C10	1.504 (16)
O4—C6	1.266 (13)	C10—H10A	0.9600
O5—H5D	0.8200	C10—H10B	0.9600
O5—H5E	0.8900	C10—H10C	0.9600
O6—C12	1.208 (13)	C11—C14	1.468 (14)
O7—C14	1.252 (13)	C11—C12	1.477 (15)
C1—C3	1.391 (16)	C11—H11	0.9800
C1—C2	1.468 (15)	C12—C13	1.513 (16)
C2—H2A	0.9600	C13—H13A	0.9600
C2—H2B	0.9600	C13—H13B	0.9600
C2—H2C	0.9600	C13—H13C	0.9600
C3—C4	1.406 (16)	C14—C15	1.520 (15)
C3—H3	0.9300	C15—H15A	0.9600
C4—C5	1.518 (16)	C15—H15B	0.9600
C5—H5A	0.9600	C15—H15C	0.9600
C5—H5B	0.9600		
			
O1—Ir1—O4	177.1 (3)	O4—C6—C7	115.2 (11)
O1—Ir1—O3	84.6 (3)	C8—C6—C7	117.6 (11)
O4—Ir1—O3	95.1 (3)	C6—C7—H7A	109.5
O1—Ir1—O2	94.2 (3)	C6—C7—H7B	109.5
O4—Ir1—O2	85.9 (3)	H7A—C7—H7B	109.5
O3—Ir1—O2	175.4 (3)	C6—C7—H7C	109.5
O1—Ir1—C11	87.6 (4)	H7A—C7—H7C	109.5
O4—Ir1—C11	95.3 (4)	H7B—C7—H7C	109.5
O3—Ir1—C11	93.5 (4)	C6—C8—C9	128.5 (11)
O2—Ir1—C11	90.9 (4)	C6—C8—H8	115.7
O1—Ir1—O5	91.6 (3)	C9—C8—H8	115.7
O4—Ir1—O5	85.5 (3)	O3—C9—C8	126.0 (11)
O3—Ir1—O5	87.3 (3)	O3—C9—C10	113.8 (10)
O2—Ir1—O5	88.2 (3)	C8—C9—C10	120.2 (11)
C11—Ir1—O5	178.8 (4)	C9—C10—H10A	109.5
C1—O1—Ir1	123.5 (7)	C9—C10—H10B	109.5
C4—O2—Ir1	122.2 (7)	H10A—C10—H10B	109.5
C9—O3—Ir1	121.2 (7)	C9—C10—H10C	109.5
C6—O4—Ir1	121.9 (8)	H10A—C10—H10C	109.5
Ir1—O5—H5D	109.5	H10B—C10—H10C	109.5
Ir1—O5—H5E	109.5	C14—C11—C12	117.6 (10)
H5D—O5—H5E	109.2	C14—C11—Ir1	112.4 (8)
O1—C1—C3	123.7 (10)	C12—C11—Ir1	108.4 (7)
O1—C1—C2	115.6 (11)	C14—C11—H11	105.8
C3—C1—C2	120.7 (11)	C12—C11—H11	105.8
C1—C2—H2A	109.5	Ir1—C11—H11	105.8
C1—C2—H2B	109.5	O6—C12—C11	123.2 (11)
H2A—C2—H2B	109.5	O6—C12—C13	119.2 (11)
C1—C2—H2C	109.5	C11—C12—C13	117.6 (11)
H2A—C2—H2C	109.5	C12—C13—H13A	109.5
H2B—C2—H2C	109.5	C12—C13—H13B	109.5
C1—C3—C4	129.1 (11)	H13A—C13—H13B	109.5
C1—C3—H3	115.5	C12—C13—H13C	109.5
C4—C3—H3	115.5	H13A—C13—H13C	109.5
O2—C4—C3	126.9 (11)	H13B—C13—H13C	109.5
O2—C4—C5	114.0 (11)	O7—C14—C11	120.8 (10)
C3—C4—C5	119.1 (11)	O7—C14—C15	117.0 (10)
C4—C5—H5A	109.5	C11—C14—C15	122.2 (10)
C4—C5—H5B	109.5	C14—C15—H15A	109.5
H5A—C5—H5B	109.5	C14—C15—H15B	109.5
C4—C5—H5C	109.5	H15A—C15—H15B	109.5
H5A—C5—H5C	109.5	C14—C15—H15C	109.5
H5B—C5—H5C	109.5	H15A—C15—H15C	109.5
O4—C6—C8	127.1 (11)	H15B—C15—H15C	109.5
			
O4—Ir1—O1—C1	96 (6)	C1—C3—C4—C5	−176.5 (12)
O3—Ir1—O1—C1	−179.7 (9)	Ir1—O4—C6—C8	2.3 (17)
O2—Ir1—O1—C1	4.8 (9)	Ir1—O4—C6—C7	179.4 (8)
C11—Ir1—O1—C1	−85.9 (9)	O4—C6—C8—C9	1(2)
O5—Ir1—O1—C1	93.2 (9)	C7—C6—C8—C9	−176.4 (13)
O1—Ir1—O2—C4	0.1 (9)	Ir1—O3—C9—C8	1.1 (16)
O4—Ir1—O2—C4	−177.0 (9)	Ir1—O3—C9—C10	−178.4 (8)
O3—Ir1—O2—C4	−76 (4)	C6—C8—C9—O3	−3(2)
C11—Ir1—O2—C4	87.7 (9)	C6—C8—C9—C10	176.9 (13)
O5—Ir1—O2—C4	−91.4 (9)	O1—Ir1—C11—C14	149.4 (8)
O1—Ir1—O3—C9	−176.0 (8)	O4—Ir1—C11—C14	−30.7 (8)
O4—Ir1—O3—C9	1.1 (8)	O3—Ir1—C11—C14	−126.1 (8)
O2—Ir1—O3—C9	−100 (4)	O2—Ir1—C11—C14	55.2 (8)
C11—Ir1—O3—C9	96.8 (9)	O5—Ir1—C11—C14	101 (17)
O5—Ir1—O3—C9	−84.1 (8)	O1—Ir1—C11—C12	−78.8 (8)
O1—Ir1—O4—C6	81 (6)	O4—Ir1—C11—C12	101.0 (8)
O3—Ir1—O4—C6	−2.7 (9)	O3—Ir1—C11—C12	5.6 (8)
O2—Ir1—O4—C6	172.7 (9)	O2—Ir1—C11—C12	−173.0 (8)
C11—Ir1—O4—C6	−96.8 (9)	O5—Ir1—C11—C12	−127 (16)
O5—Ir1—O4—C6	84.2 (9)	C14—C11—C12—O6	30.9 (17)
Ir1—O1—C1—C3	−6.4 (16)	Ir1—C11—C12—O6	−98.0 (11)
Ir1—O1—C1—C2	172.5 (8)	C14—C11—C12—C13	−146.7 (11)
O1—C1—C3—C4	2(2)	Ir1—C11—C12—C13	84.4 (11)
C2—C1—C3—C4	−176.5 (13)	C12—C11—C14—O7	135.2 (12)
Ir1—O2—C4—C3	−3.7 (17)	Ir1—C11—C14—O7	−97.9 (11)
Ir1—O2—C4—C5	176.3 (7)	C12—C11—C14—C15	−45.0 (16)
C1—C3—C4—O2	3(2)	Ir1—C11—C14—C15	82.0 (12)

### Hydrogen-bond geometry (Å, °)

**Table:**

*D*—H···*A*	*D*—H	H···*A*	*D*···*A*	*D*—H···*A*
O5—H5D···O7^i^	0.82	2.24	2.706 (11)	116
O5—H5E···O6^ii^	0.89	2.11	2.715 (11)	125

Symmetry codes: (i) *x*, *y*+1, *z*; (ii) *x*+1, *y*, *z*.
